# PARP inhibitors

**DOI:** 10.1186/s13053-014-0024-8

**Published:** 2015-01-17

**Authors:** Maheen Anwar, Hafiz Muhammad Aslam, Shahzad Anwar

**Affiliations:** Dow Medical College, Dow University of Health Sciences, Karachi, Pakistan; Final year student of Dow Medical College, Dow University of Health Sciences, Karachi, Pakistan

**Keywords:** Poly (ADP-ribose) polymerases, Nicotinamide, Rucaparib

## Abstract

Poly (ADP-ribose) polymerases, abbreviated as PARPs, are a group of familiar proteins that play a central role in DNA repair employing the base excision repair (BER) pathway. There about 17 proteins in this family out of which the primary nuclear PARPs are PARP-1, PARP-2, PARP-3, and tankyrases 1 and 2 (PARP-5a and -5b) .The PARP family members are known to engage in a wide range of cellular activities, for example, DNA repair, transcription, cellular signaling, cell cycle regulation and mitosis amongst others. The chief functional units of PARP-1 are an amino terminal DNA binding domain (DBD), a central auto modification domain (AMD), and a carboxyl-terminal catalytic domain (CD). PARP inhibitors are currently undergoing clinical trials as targeted treatment modalities of breast, uterine, colorectal and ovarian cancer. This review summarizes current insights into the mechanism of action of PARP inhibitors, its recent clinical trials, and potential next steps in the evaluation of this promising class of anti-cancer drugs.

## Findings

Poly (ADP-ribose) polymerases, abbreviated as PARPs, are a group of familiar proteins that play a central role in DNA repair employing the base excision repair (BER) pathway. These nuclear proteins possess enzymatic and scaffolding properties and govern the repair of single strand breaks in DNA [1]. A true poly(ADP-ribose) polymerase (PARP) can transfer the first ADP-ribose moiety from nicotinamide adenine dinucleotide (NAD+) to an acceptor protein (preferably to glutamate or lysine residues) and can sequentially add multiple ADP-ribose units to the preceding ones to form poly(ADP-ribose) (pADPr) chains. There about 17 proteins in this family out of which the primary nuclear PARPs are PARP-1, PARP-2, PARP-3, and tankyrases 1 and 2 (PARP-5a and -5b). The PARP family members are known to engage in a wide range of cellular activities, for example, DNA repair, transcription, cellular signaling, cell cycle regulation and mitosis amongst others [2-6].

Environmental exposures and cell replication result in DNA damage that is repaired by a variety of mechanisms, including base excision repair (BER), mismatch repair (MMR), nucleotide excision repair (NER), single strand annealing (SSA), homologous recombination (HR), and nonhomologous end joining (NHEJ). Poly (ADP-ribose) polymerases (PARPs) are a family of proteins involved in DNA repair that utilize the BER pathway and share enzymatic and scaffolding properties. PARP1 and PARP2 are the best studied members of this family of enzymes. PARP1 has three domains that are responsible for DNA-binding, automodification, and catalysis. DNA cleavage results in the recruitment and binding of PARP1 to the site of damage, with an increase in its catalytic activity, and the formation of long, branched, poly (ADP-ribose) (PAR) chains. PAR has a net negative charge that promotes recruitment of DNA repair proteins involved in the BER pathway to the site of DNA damage, and facilitates removal of PARP1 from damage sites, allowing access to other repair proteins. Apart from its role in BER, PARP1 has been implicated in the HR and NHEJ pathways, suggesting a broader role for this enzyme family in the overall DNA repair process. PARP1 and PARP2 are the ones extensively studied and well known to be stimulated by DNA damage [7-9]. The discovery of their existence was made in 1963, and since then over 40 years of extensive research efforts has brought forth the fruitful results of their potential as therapeutic agents for cancer [8].

Characterized best amongst the PARP super family members; PARP1 has an integrated structure based on many independently folded domains out of which three are the most important. The chief functional units of PARP-1 are an amino terminal DNA binding domain (DBD), a central auto modification domain (AMD), and a carboxyl-terminal catalytic domain (CD) [3,5]. PARP1 is over expressed in a variety of cancers. Its expression has been linked with prognosis of cancers, most notably breast cancer [10]. PARP1 and its product, PAR, can respond to a variety of endogenous and exogenous stress signals including those generated by oxidative, genotoxic, thermal, oncogenic, metabolic and inflammatory stresses. These responses trigger pathological conditions such as cancer, inflammation related diseases, autoimmune diseases, neurodegenerative diseases and metabolic stresses. PARP inhibitors can therefore be followed upon as a therapeutic solution to these pathologic states [11].

PARP inhibitors in clinical development imitate the nicotinamide moiety of nicotinamide adenine dinucleotide, and bind to the enzyme’s catalytic domain, inhibiting auto modification and subsequent release of the enzyme from the site of DNA damage. Simultaneously, they also impede access of other repair proteins to the site of DNA damage [9].

PARP inhibitors are currently undergoing clinical trials as targeted treatment modalities for cancer. Environmental and genetic stressors that disrupt the cell cycle are vital to the etiology and progression of cancer. Henceforth, PARP-1 is an indispensible role player in tumour cell development and PARP-1 targeted therapy can positively predict the outcome in cancer therapy. Clinical trials have been undertaken to assess the safety and efficacy profiles of PARP inhibitors for management of breast, uterine, colorectal and ovarian cancers [1].

The efficacy of these drugs may be due to the phenomenon of synthetic lethality. This phenomenon targets cells deficient in one DNA repair pathway by inhibiting another. Tumor cells in which the BRCA gene is mutated are unable to repair DSBs by HRR and hence are reliant on other DNA repair pathways, such as BER pathway, which can preclude the development of double stranded breaks DSBs in DNA. Inhibiting PARP activity and subsequent BER pathway in such cells causes the unrepaired single stranded breaks to develop into double stranded breaks and leads to cell death. Such ‘synthetic lethality’ carries implications for the development of anti-cancer drugs [12]. Some tumors possess faulty Homologous Recombination (HR) mechanisms that arise from acquired flaws in the HR pathway rather than germ line BRCA mutations- this property being known as BRCAness. It can occur as a consequence of epigenetic modifications of BRCA 1/2 and/or mutations in various proteins crucial to HR pathways such as RAD51, RAD54, DSS1, RPA1, ATM, CHK2 and PTEN and has been related to several malignancies including triple-negative breast cancer and sporadic serous ovarian cancer [13-15].

Inhibiting PARP activity uncovers potential of PARP inhibitors as promising candidates for cancer therapy, particularly in BRCA1/2 mutated cancers, alone or in combination with cytotoxic drugs [12,16,17]. p53-deficient breast cancer cells treated with a PARP Inhibitor happen to lose resistance to an apoptosis promoting, clinically active antitumor agent called doxorubicin [18]. As we established previously, PARP inhibitors were recently developed on the rationale of synthetic lethality, however this concept was well illustrated by Byrant et all and Farmer et all in 2005. They demonstrated that, breast cancer cells containing germ line mutations in BRCA1 and BRCA 2 genes become sensitized to PARP inhibitors in a PARP1 dependant manner and lose sensitivity to PARP inhibition on regaining BRCA2 functionality [19]. A clinical trial of a PARP inhibitor drug, Olaparib, based on the approach of synthetic lethality, has given successful results (minimal side effects with safely administrable doses) in breast cancers containing BRCA1/2 mutations [16].

The first PARP inhibitor, Nicotinamide, was identified in 1971. Second generation drugs were recognized subsequently. Although agents having selective activity upon PARP-1 and PARP-2 have been identified, most PARP inhibitors are non-selective, affecting PARP-1 as well as PARP-2 [1].

As of the present day, PARP inhibitors have been explored either as sole agents in BRCA-1/2 depleted cancer or in cancers possessing BRCA-ness, or combined with other DNA damaging moieties such as ionizing radiation in an expansive array of cancers. There are a total of nine drugs in the hierarchy of phases of drug development [20-22].

**AZD2281** (Olaparib) is a small molecule oral PARP1/2 and TNKS inhibitor with the commonly observed toxicities of nausea, vomiting, diarrhea and fatigue [16,23]. It is currently undergoing phase I and phase II clinical trials as single agent or in combination therapy with chemotherapy in different cancers like breast, ovarian and colorectal cancers [24].

**AG-014699** (PF-01367338) Rucaparib is an intravenous PARP inhibitor with no significant toxicities when used alone. It was examined in combination with temozolomide in advanced solid tumors [25]. When this combination was used as a first line intervention in patients with metastatic melanoma, bone marrow toxicity was observed [26]. Currently it is being evaluated in Phase II trials as solo agent in BRCA associated breast and ovarian cancers and in Phase I trials in combination with chemotherapy for advanced solid malignancies [24].

**ABT-888** (Veliparib) is an oral PARP-1/2 inhibitor with decent oral bioavailability and apparent CNS penetration [20]. It is currently being explored in Phase I and II Trials in combination with radiation and chemotherapy, respectively, in various cancer types including breast cancer, colorectal cancer, glioblastoma multiforme and melanoma [24].

**MK-4827 (niraparib)**, an orally available compound, demonstrating strong PARP-1 and PARP-2 inhibitory activity has the side effects of nausea, vomiting, fatigue and thrombocytopenia [27]. It is currently enrolled in Phase I trials as a single agent or in combination regimens with carboplatin in advance solid cell malignancies [24].

**CEP-9722** is an oral PARP-1/2 inhibitor and a pro-drug of CEP-8983. It has the capability of sensitizing tumor cells in glioblastoma, colon cancer, neuroblastoma and rhabdomyosarcoma to TMZ, irinotecan and radiation without augmenting myelosuppression [28]. It is currently under trial as single agent and in combination therapy with Temoolomide in advanced solid malignancies [23].

**E7016** (GPI-21016) (formerly known as GPI-21016) is an orally bioavailable PARP inhibitor that can enhance tumor radio sensitivity and also synergies with temozolmide and radiation to potentiate their effect [29]. It is also enrolled in an ongoing Phase I trial which is exploring its efficacy in combination with temozolomide in advanced solid tumors and gliomas [23].

Other orally bioavailable potent PARP inhibitor agents include INO-1001, MP-124 and LT-00673 which are also undergoing Phase I clinical development trials [20].

Main function of radiotherapy is to produce DNA strand breaks, causing severe DNA damage and leading to cell death. Radiotherapy has the potential to kill 100% of any targeted cells, but the dose required to do so would cause unacceptable side effects to healthy tissue. Radiotherapy therefore can only be given up to a certain level of radiation exposure. Combining radiation therapy with PARP inhibitors offers promise, since the inhibitors would lead to formation of double strand breaks from the single-strand breaks generated by the radiotherapy in tumor tissue with BRCA1/BRCA2 mutations. This combination could therefore lead to either more powerful therapy with the same radiation dose or similarly powerful therapy with a lower radiation dose.

It is astonishing to know that the utility of PARP inhibitors as therapeutic candidates is not limited to their efficacy in treating malignancies. Over the decade, they have emerged with a spectrum of actions that incorporates treating other conditions such as cardiovascular diseases, stroke, metabolic disorders, diabetes, and autoimmunity [30-34].
